# The Metropolitan Asylums Board of London and Its Work.—1

**Published:** 1900-09-22

**Authors:** 


### The Institutional Workshop.

### THE METROPOLITAN ASYLUMS BOARD OF LONDON AND ITS WORK.—1.

By a Correspondent.

Under the above title an interesting little pamphlet has been issued by the Metropolitan Asylums Board, by way of guide to the exhibit in the Social Science section of the Paris Exhibition, demonstrating the provision made in London for (1) the treatment of infectious sick, (2) the care of the mentally deficient, and (3) the education and training of certain classes of children who become chargeable to the State.

The chief work of this important metropolitan body comes under the first head, and it is doubtless true that "so far as concerns hospital accommodation for the infectious sick the work of the Board is without parallel throughout the world." Its general expenditure already exceeds £700,000 per annum. It has under its control an area of 121 square miles, with a population of 4,589,129 (Midsummer, 1900); "13 large hospitals, containing over 6,000 beds; six land ambulance stations, with accommodation for 138 coachmen, 105 horses, and 135 vehicles; a river ambulance service, comprising three wharves and four ambulance steamboats; four asylums, containing nearly 6,000 imbecile patients; a training ship for 600 boys; several schools for certain other classes of poor children; and the provision of other establishments is in contemplation." Created in 1867, the Asylums Board is now composed of 72 members, 54 of whom are elected by the various Metropolitan boards of guardians, and 18 are nominated by the Local Government Board. Prior to that date organised provision for the isolation and treatment of persons suffering from infectious illness there was none, and the only hospitals for the special reception of such cases were the London Fever Hospital, at Islington, and the Small-pox Hospital, at Highgate, both voluntary institutions. The following table will show what has been accomplished by the Board since its creation, in spite of very grave difficulties in securing suitable sites:—

Hospitals Erected and in Use or in Course of Construction in 1900, and the Date when Those in Use were Opened.

| Hospital. | Date of Opening. | Number of Beds. | |
|---|---|---|---|
| *For acute fever and diphtheria cases.* | | | |
| 1. North-Western | January, 1870 | 460 (*a*) | |
| 2. South-Western | January, 1871 | 366 | |
| 3. Eastern | February, 1871 | 362 | |
| 4. Western | March, 1877 | 454 | |
| 5. South-Eastern | March, 1877 | 435 | |
| 6. North-Eastern | October, 1892 | 386 (*b*) | |
| 7. Fountain | October, 1893 | 402 | |
| 8. Brook | August, 1896 | 488 | |
| 9. Park | November, 1897 | 548 | |
| 10. Grove | August, 1899 | 520 | |
| | | | 4,421 |
| *For convalescent cases.* | | | |
| 1. Northern | September, 1877 | 764 (*c*) | |
| 2. Southern (in course of construction) | | 700 | |
| | | | 1,464 |
| Total | | | 5,885 |
| *For acute small-pox cases.* | | | |
| 1. Floating hospital | July, 1881 | 300 | |
| 2. Permanent land hospital (in course of construction) | | 400 | |
| | | | 700 |
| *For convalescent cases.* | | | |
| 1. Gore Farm | October, 1890 | | 1,192 |
| Total | | | 1,892 |

(*a*) Reconstructed in permanent materials since 1870. (*b*) In process of reconstruction in permanent materials. (*c*) Includes a wooden hut for 100 beds, erected during an epidemic of scarlet fever.

At first accommodation was intended for pauper cases only, but experience and common sense quickly demonstrated the necessity for a far wider interpretation of the needs of London in the interests of the public health, and it soon became a matter of importance to induce as many as possible of those who could not with safety and efficiency be nursed at home, to avail themselves of the accommodation offered. The remission of the civil disabilities at first consequent upon admission to the hospitals, with their stigma of pauperism, and the free admission and treatment of every victim to an infectious disease, subject only to a medical certificate, promptly brought about the desired result. From 7,809 fever patients admitted in 1891 to the hospitals under the control of the Managers of the Metropolitan Asylums District, the numbers have risen to 25,094 in 1899; these include sufferers from scarlet fever, diphtheria, enteric fever, and typhus fever.

It is impossible here to enter into the very interesting details of the excellent ambulance service maintained by the Board, by means of which patients are transported with a maximum of comfort and a minimum of risk at any time, by land or water, to the hospitals or small-pox ships. Every ambulance is accompanied by an experienced and fully-trained nurse, these being supplied by the staffs of the various hospitals, a certain number told off each day for the duty.

The treatment of small-pox cases is now confined wholly to the floating hospitals, moored in the river at an isolated part some 17 miles below London Bridge, it having been found in practice undesirable to aggregate large numbers of patients suffering from this disease in populous districts. A permanent hospital for small-pox is about to be erected, how ever, on land near the ships but far from any inhabited building.

Looking back on the general result of the system created under the Board, it is held that the enormous reduction in mortality from infectious sickness during recent years is in no small measure due to it. It can certainly with justice be claimed that "to the improved ambulance arrangements, and the isolation of infectious patients in special and well-designed hospitals is largely due to the annual saving of 6,800 lives which, under former conditions, would have been lost; and, further, that small-pox has so far been prevented from regaining its former prevalence in London."

Before leaving this department of the Board's work it should be noted that, amongst the Paris exhibits, models of the North-Eastern Temporary Hospital (architects, Messrs. A. and C. Harston, F.R.I.B.A.), and of the Brook hospital, Shooter's Hill (architect, Mr. T. W. Aldwinckle, F.R.I.B.A.), found prominent places. The floating Hospitals and ambulance steamers and carriages were also shown, and the exhibit was awarded a grand prize.

Turning now for a moment to the useful work carried out by the Metropolitan Asylums Board with regard to the care of imbeciles and idiots, as distinct from lunatics, we find that until the institutions built by the Managers at Caterham and Leavesden were opened, in 1870, the workhouse was the only refuge available for these afflicted beings, both children and adults. Schools have been added where the children are subjected to special courses of education and manual training, and a further classification is now being carried out by which those young people who are found to be improvable are not drafted into the ordinary adult asylums, but consigned to an industrial colony, where "the training given in the schools can be continued and utilised in proper workshops, and where much of the works and repairs required at other institutions of the Board, such as basket and mat making, carpentering, tailoring, upholstering, and shoe-making, and possibly printing and weaving, may be carried on." This part of the scheme is only now being completed, but the Managers rightly attach much importance to it, believing in this way a portion of their patients may become at least partially self-supporting, and so useful and happy members of the community.

In 1876 a further extension of its powers and duties was placed upon the Managers, as representing all the metropolitan boards of guardians, and the control of the training-ship "Exmouth," for the training for sea service of boys from district and workhouse schools was placed in their hands. This departure has proved eminently successful, a very large number of the boys thus trained having passed into the Royal Navy.

The latest addition to the Metropolitan Asylums Board's responsibilities has been that of "providing for certain exceptional classes of children," those suffering from ophthalmia, contagious diseases of skin or scalp, those requiring special treatment during convalescence or sea air, those of feeble intellect, and those ordered to a workhouse or asylum by a magistrate. Schools for these children on the "cottage home" principle are being built, seaside homes have been secured, and a scheme mapped out which should insure the best possible result from every point of view.

